# Mapping illegal trade routes of live cheetahs from the Horn of Africa to the Arabian Peninsula

**DOI:** 10.1111/cobi.14412

**Published:** 2024-10-25

**Authors:** Paul H. Evangelista, Nicholas E. Young, Darin K. Schulte, Patricia D. Tricorache, Matthew W. Luizza, Sarah M. Durant, Kelly W. Jones, Nicholas Mitchell, Tomas Maule, Abdullahi H. Ali, Redae T. Tesfai, Peder S. Engelstad

**Affiliations:** ^1^ Natural Resource Ecology Laboratory Colorado State University Fort Collins Colorado USA; ^2^ Division of International Conservation U.S. Fish and Wildlife Service Falls Church Virginia USA; ^3^ Institute of Zoology Zoological Society of London London UK; ^4^ The Africa Range‐Wide Cheetah Conservation Initiative Zoological Society of London London UK; ^5^ Department of Fish, Wildlife and Conservation Ecology New Mexico State University Las Cruces New Mexico USA; ^6^ Torrid Analytics Nairobi Kenya; ^7^ Hirola Conservation Program Garissa Kenya

**Keywords:** *Acinonyx jubatus soemmeringii*, decision support tool, habitat suitability index, network modeling, species distribution models, wildlife trafficking, herramienta de apoyo para las decisiones, índice de idoneidad del hábitat, modelos de distribución de especies, redes de modelos, tráfico de fauna, Acinonyx jubatus soemmeringii

## Abstract

Less than 7000 cheetahs (*Acinonyx jubatus*) persist in Africa. Although human–wildlife conflict, habitat degradation, and loss of prey are major threats to cheetah populations, illegal trade in live cubs for pets may have the most significant impact on populations in the Horn of Africa. We developed a novel, stepwise decision support tool to predict probable trafficking routes by leveraging the power of distinct modeling approaches. First, we created a cheetah habitat suitability index (HSI) to determine where source cheetah populations may occur. We then created a trafficking network model linking known and predicted cheetah populations with documented destinations in the Arabian Peninsula. A significant area in Eastern Ethiopia and Northern Somalia was estimated to harbor undocumented cheetahs. When these predicted populations were used as a supply source, the trafficking network model showed multiple routes passing through Somaliland and across the Gulf of Aden to Yemen, supporting the notion that undocumented cheetahs may be supplying pet market demands. Though we demonstrate how our decision support tool can inform law enforcement, conservation strategies, and community engagement, we caution that our results are not fully validated due to limited accessibility, alternative trafficking routes, and the cryptic nature of illegal wildlife trade.

## Abstract (es)

Mapeo de las rutas de tráfico ilegal de chitas vivas del Cuerno de África a la Península Arábiga

**Resumen:**

En África hay menos de 7,000 chitas (Acinonyx jubatus). Aunque las principales amenazas para las poblaciones de chitas son el conflicto humano‐fauna, la degradación del hábitat y la pérdida de presas, el mercado ilegal de cachorros como mascotas puede que tenga el impacto más significativo en las poblaciones del Cuerno de África. Desarrollamos una novedosa herramienta por pasos para la toma de decisiones para predecir las probables rutas de tráfico aprovechando el poder de las distintas estrategias de modelación. Primero creamos un índice de idoneidad del hábitat (IIH) de las chitas para determinar en dónde podrían encontrarse las poblaciones de origen de chitas. Después creamos un modelo de redes de tráfico que conecta las poblaciones conocidas y pronosticadas con los destinos documentados en la Península Arábiga. Estimamos que un área significativa en el este de Etiopía y en el norte de Somalia albergaba chitas de manera ilegal. Cuando usamos estas poblaciones pronosticadas como fuente de suministro, el modelo de redes de tráfico mostró varias rutas que atraviesan Somalilandia y el Golfo de Adén hacia Yemen, lo que apoya la noción de que las chitas ilegales podrían estar cumpliendo las demandas del mercado de mascotas. Aunque demostramos que nuestra herramienta de apoyo para las decisiones puede informar al orden público, también advertimos que nuestros resultados no están del todo validados debido a la accesibilidad restringida, rutas alternativas de tráfico y la naturaleza críptica del mercado ilegal de fauna.

## INTRODUCTION

The decline of free‐ranging cheetah (*Acinonyx jubatus*) populations in Africa has been observed since the 1960s (Myers, 1975). Once widespread throughout the continent, estimates suggest that only 6500 adult and adolescent cheetahs remain in Africa and inhabit only 13% of their former range (Durant et al., 2017). In East Africa, approximately 2290 cheetahs are thought to remain in the wild and are concentrated in 15 fragmented subpopulations across Ethiopia, Kenya, Tanzania, South Sudan, and Uganda (Durant et al., 2017). Human–wildlife conflict, habitat loss and fragmentation (exacerbated by low density and large home ranges of cheetah), loss of prey due to grazing competition with livestock and unsustainable bushmeat extraction, and the trade of skins and live animals have been, and continue to be, direct threats to cheetah populations (Durant et al., 2022; Nowell, 2014; Tricorache et al., 2018). However, the International Union for Conservation of Nature (IUCN) Red List assessment states that “cheetah populations in the Horn of Africa, close to the Somali border, are likely to suffer the strongest impacts of the illegal trade in live animals in East Africa” (Durant et al., 2022).

Although cheetahs have been used as companions or hunting assistants to nobles and kings, particularly in Asia and India, for over 3000 years (Allsen, 2006; Divyabhanusinh, 1995; Walker, 2001), the trade of live cheetahs, primarily for pets in the Arabian Peninsula, has occurred largely unnoticed until the last decade. Nowell (2014), in a report commissioned by the Convention on International Trade of Endangered Species of Wild Fauna and Flora (CITES), documented more than 40 live cheetah confiscations from 2011 to 2013, mostly in Somaliland, the self‐declared autonomous state internationally recognized as de jure part of Somalia. Even more alarming is a recent report documenting more than 1880 cases of seized or marketed live cheetahs and parts from 2010 to 2019 (Tricorache et al., 2021). This report shows that multiple national laws or CITES regulations related to the capture, killing, trade, and transport of wildlife have been violated and that an estimated 4000 cheetahs (live or parts and derivatives) were affected. Of these, 3645 (91%) were live cheetahs, mostly cubs. The vast majority were taken from the Horn of Africa and destined for wealthy countries in the nearby Arabian Peninsula, where pet cheetahs are in high demand and considered symbols of status, wealth, and prestige for their owners (Mitchell & Durant, 2017; Tricorache et al., 2018). Furthermore, data collected from 2020 to 2022 (P.D.T., unpublished data) suggest that the illegal cheetah trade has grown significantly; annual recorded incidences increased by over 60% relative to the annual average of the previous 10 years. Confiscations remained consistent at <10% of the estimated trade volume. This rate of offtake, when the best estimates suggest only around 300 individual cheetahs still survive in Horn of Africa countries (Durant et al., 2017), is unsustainable and threatens endemic cheetah populations, particularly the rare northeastern African subspecies *A. jubatus soemmeringii*, which was recently classified by the IUCN Red List of Threatened Species as endangered (Durant, Broekhuis, et al., 2023).

**FIGURE 1:**
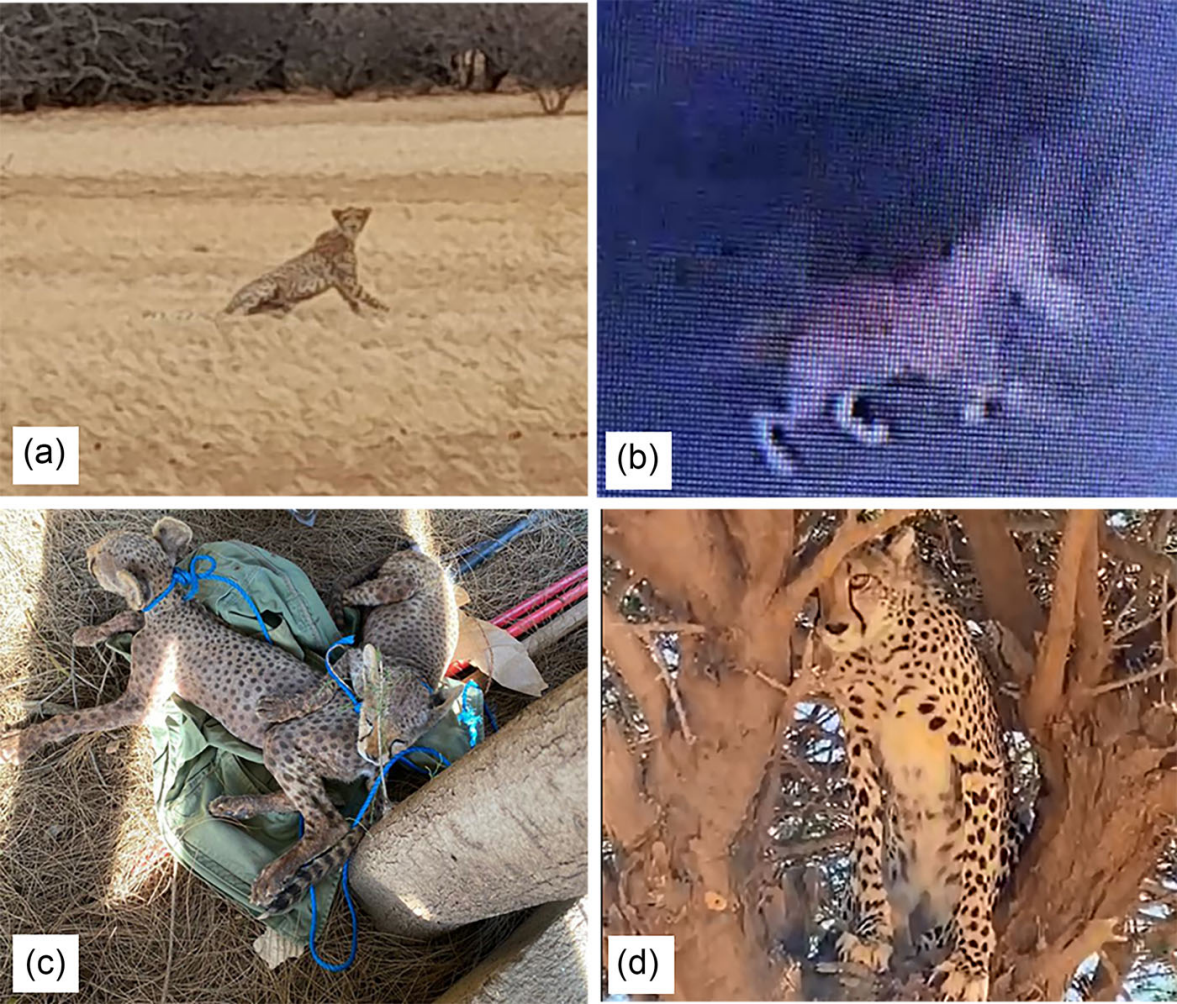
(a) An adult female cheetah with a juvenile observed in July 2023 in eastern Ethiopia (photograph courtesy of Bureau of Environment Protection and Rural Land Administration, Somali Regional State); (b) cheetah on the outskirts of a town in northern Djibouti (photograph courtesy of Ministère de l'Environnement et du Développement Durable, Djibouti); (c) cheetah cubs confiscated in April 2023 in the Somali Regional State, Ethiopia (photograph courtesy of the Bureau of Environment Protection and Rural Land Administration, Somali Regional State); and (d) an adult cheetah captured in a tree and then shot by locals in Sanaag, Somaliland, in December 2023 (photograph by courtesy of Puntland's Ministry of Environment, Range and Climate Change).

Information on the trafficking of cheetahs is sparse because much of the trade takes place in areas of political insecurity, where access for researchers and investigators is difficult and the extent of the source of live cubs that supply the pet markets remains uncertain. However, work on the Ethiopian side of the border with Somaliland (Abdella et al., 2023) shows that area represents an important source for the trafficking of cheetah cubs. Moving live cheetah cubs from these areas to the Arabian Peninsula would presumably be difficult because young cubs are extremely vulnerable. Much of the Horn of Africa, particularly the countries bordering the Gulf of Aden, have not been adequately surveyed for cheetah presence or other wildlife in decades due to conflict, insecurity, or the sheer remoteness of these regions (Agnelli et al., 1990; Durant, Breitenmoser, et al., 2023). In 2017, interviews with 200 agropastoralists across Somaliland showed the occurrence and commonality of 53 wildlife species and their approximate location (Evangelista et al., 2018). Of these, 177 respondents reported that cheetahs were found in their immediate vicinity, either commonly (*n* = 68) or rarely (*n* = 109). Marker et al. (2023) reported similar results of cheetah presence in the Awdal region of western Somaliland in 2021 and 2022. Photographic evidence of cheetahs in and adjacent to regions where the status of the species has been suspected, but not confirmed, has surfaced. In 2021, a pilot photographed a mother and 3 juveniles from the air in the Somali Regional State of Ethiopia (B. Polo, personal communication 2023), and in 2022, a team of researchers recorded an adult cheetah on a camera trap in Djibouti (Murgatroyd et al., 2023). Additional photographs of cheetahs, confiscated cubs, and individuals illegally killed confirm that they persist in regions where their presence is thought to be possible and support the notion that a viable number of cheetahs could remain undetected where information is scant (Figure 1).

Illegal wildlife trade (IWT) is valued at more than US$20 billion annually and is a part of the broader category of environmental crimes, which, by some estimates, constitute the fourth largest global crime category, following drug trafficking, counterfeit crimes, and human trafficking (UNEP, 2016; UNODC, 2020). Moreover, IWT may often operate alongside legal wildlife trade, with a global annual estimated value of US$119 billion (van Uhm, 2016), and products from IWT may be laundered into these licit wildlife supply chains because of corruption and gaps in enforcement. In light of the threats to species survival, the high revenues generated, and implications for global health and security, IWT continues to receive heightened attention from the international community, for example, CITES (e.g., Resolution Conf. 11.17 [Rev.CoP18] 2016), the United Nations General Assembly (e.g., A/RES/73/343, 2019), and national legislation (e.g., U.S. P.L. 114–231, 2016). This attention acknowledges the growing need for an improved understanding of IWT supply and demand, including illicit supply chains. There is a need for an improved understanding of IWT dynamics through interdisciplinary research, including leveraging distinct disciplinary frameworks and diverse data streams (Gore et al., 2023; Hilend et al., 2023; Keskin et al., 2023).

Today, the demand for live cheetah remains high. Small populations in areas of East Africa are facing the highest threat, due to their reduced numbers and the region's proximity to wealthy nations of the Arabian Peninsula (Mitchell & Durant, 2017; Tricorache et al., 2018). Wild cubs destined for these markets are taken from their mothers and transported on small boats across the Gulf of Aden to isolated shores in Yemen (Tricorache & Stiles, 2021). In Yemen, dealers hide them near animal markets or private homes and then distribute them over land throughout the Gulf States after negotiations with intermediaries or end buyers. Of the 3645 live cheetahs reported as in trade by Tricorache et al. (2021), 3533 (97%) were recorded in East Africa and the Arabian Peninsula. Five countries in the Arabian Peninsula (i.e., Kuwait, Qatar, Saudi Arabia, United Arab Emirates, and Yemen) and 4 cheetah‐range countries in the Horn of Africa (i.e., Ethiopia, Kenya, Somalia, and Somaliland) accounted for 95% of all live cheetahs in illegal trade incidents recorded globally. At the source, a young cheetah cub can sell for as little as US$80. Their value increases as they reach Yemen and other nations in the Arabian Peninsula; prices range from US$1890 to US$8100 for intermediaries, and up to US$30,000 for end buyers (Tricorache & Stiles, 2021). These economic incentives and the limited enforcement to combat the illegal trade continue to drive the capture and theft of cheetah cubs from East Africa to the Arabian Peninsula.

Our goal was to identify the likely trafficking routes of illegal live cheetah trade from the Horn of Africa to the Arabian Peninsula and determine priority areas for targeted intervention by law enforcement and public awareness and engagement campaigns. Using a novel stepwise process, we developed species distribution models (SDMs) for key cheetah prey species and combined them into a prey richness layer. This layer was combined with other environmental variables to create an expert‐informed habitat suitability index (HSI) of cheetahs. We used a spatially explicit trafficking network model to identify and rank potential transportation routes between sources and destinations. The trafficking network model was used to test 2 source scenarios: areas where cheetahs occur and areas identified by our HSI.

## METHODS

### Study area

**FIGURE 2:**
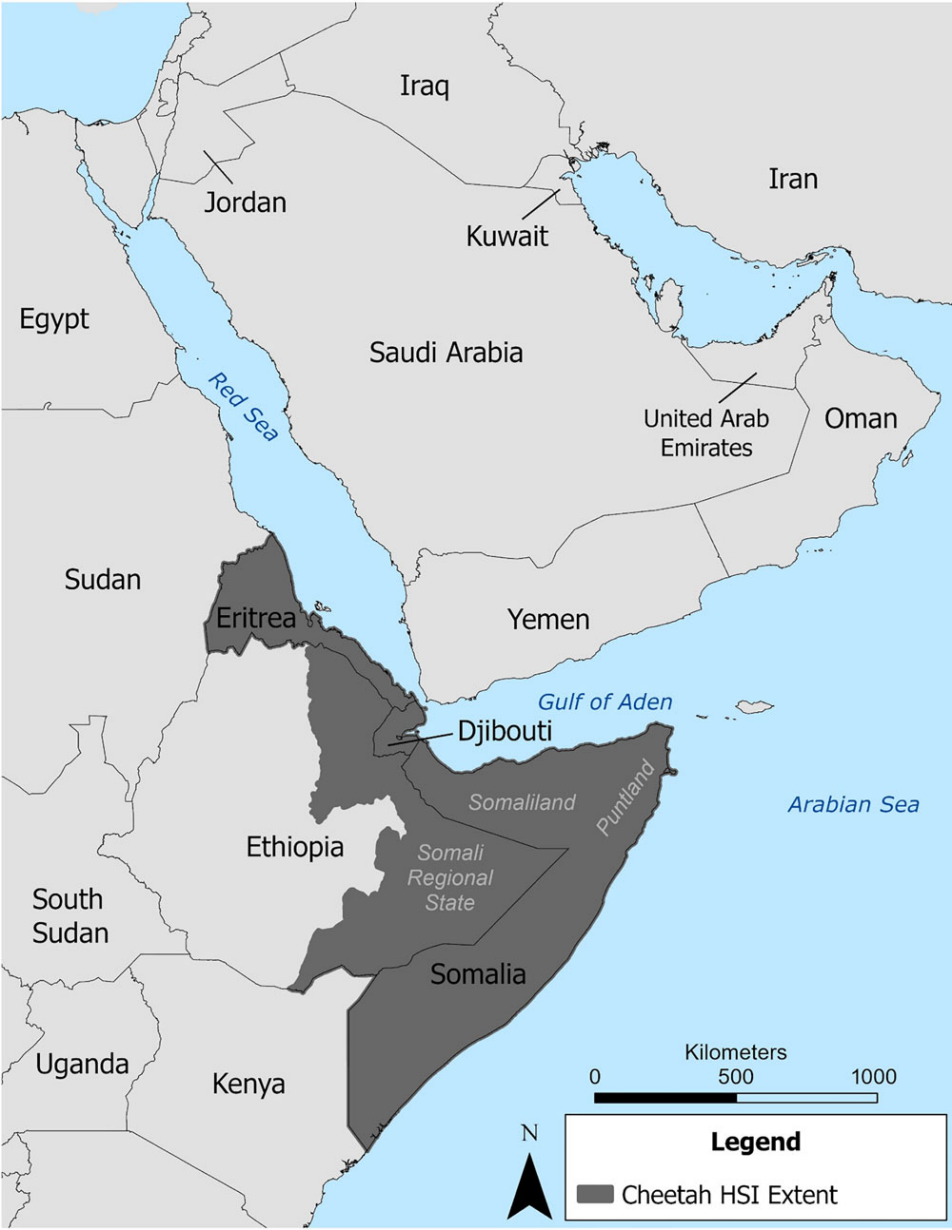
Study area including Arabian Peninsula and Horn of Africa, and the extent of the cheetah habitat suitability index (HSI).

The geographic extent of our trafficking network models included 23 countries and states from East and Northeast Africa (Burundi; Djibouti; Egypt; Eritrea; Ethiopia; Kenya; Rwanda; Somalia, including the self‐declared autonomous Republic of Somaliland; South Sudan; Sudan; Tanzania; and Uganda) and the Arabian Peninsula. We also included the Persian Gulf countries and their neighbors: Bahrain, Kuwait, Israel, Oman, Qatar, Saudi Arabia, the United Arab Emirates, Yemen, State of Palestine, and southern Iraq and Jordan (Figure 2). Some of these countries were included because they are traversed by probable trafficking routes. Others were included because they were origin or destination countries of trafficked cheetahs. A second and smaller study area, for which we developed the SDMs of prey species and the HSI of potential cheetah range, included Djibouti, Eritrea, Eastern Ethiopia, and Somalia (including Somaliland). We focused on this region because the area has similar landscapes, the status or occurrence of cheetahs is unknown, resident populations are small and require assessment, or resident populations are believed to have been extirpated (Durant et al., 2022).

### Species distribution modeling of prey

Habitat suitability and SDMs have become important tools for prioritizing conservation interventions (Guisan et al., 2013) and have the added strength of relying on data that are more readily available for species and regions that are undersurveyed or difficult to access. These models can be used to guide more targeted survey efforts to confirm presence, make effective use of limited resources, and identify and prioritize areas for conservation interventions (Evangelista et al., 2016). SDMs have been used to guide cheetah conservation strategies and management interventions in Kenya (Kuloba et al., 2015), Iran (Shams‐Esfandabad et al., 2021), Somaliland (Evangelista et al., 2018), and Zimbabwe (Tagwireyi et al., 2020). However, these efforts generally rely on occurrence locations, which are few for cheetahs in most of the Horn of Africa due to restricted access.

To compile a potential prey availability and richness layer for the cheetah HSI, we generated SDMs for multiple prey species with maximum entropy (Maxent 3.3.3k) modeling techniques. Maxent is an established correlative (i.e., machine learning) method with an algorithm that relates occurrence data and environmental conditions to estimate habitat suitability based on principles of maximum entropy (Ahmadi et al., 2023; Phillips & Dudik, 2008; Phillips et al., 2004).

Occurrence data of potential prey species for cheetahs were sourced from surveys conducted in the region that focused on the large fauna (i.e., Funaioli & Simonetta, 1966; Yalden et al., 1984, 1986). Although these observations are from many years ago, they represent the most recent and comprehensive surveys of mammals from the region. Independent surveys conducted in 1999–2000, 2010, 2015, and 2016–2017 on portions of the study area confirm the persistence of many of these species (Evangelista et al., 2018; Gedow et al., 2017; Künzel et al., 2000; Mallon & Jama, 2015). Seven species had an adequate number of occurrences available (>100 records) to develop models and were likely to represent the bulk of cheetah prey in the region: Speke's gazelle (*Gazella spekei*), Beira (*Dorcatragus megalotis*), northern gerenuk (*Litocranius walleri*), Clarke's gazelle (*Ammodorcas clarkei*), Soemmerring's gazelle (*Nanger soemmerringii*), Guenther's dik dik (*Madoqua guentheri*), and Phillip's dik dik (*Madoqua saltiana*).

For the environmental variables to inform the prey SDMs, we compiled spatial data that represented climatic conditions, land‐cover type, human modification, and topography (Appendix S1). Categorical land‐cover data from the European Space Agency Climate Change Initiative Land Cover Team (ESA CCI, 2017) were converted to continuous variables representing the percentage of each land‐cover type within 1 km^2^. All environmental variables were resampled to 1‐km resolution, and we removed one of each pair of highly correlated variables (Spearman, Pearson, or Kendall correlation coefficients |*r*| > 0.7) (Dormann et al., 2013). Maxent models were developed using default values, except that we modified the beta multiplier for models that were overfitted because an overfit model has a test and train area under the receiver operating characteristic curve (AUC) difference of >0.5 (Jarnevich et al., 2015).

Bias is important to consider when using opportunistic occurrence data in Maxent models (Kramer‐Schadt et al., 2013; Phillips et al., 2009). To help address this, we used background points that represented locations of all other species in the data sets compiled for the occurrence data. We used the threshold calculated by maximizing sensitivity plus specificity divided by 2 to create presence and absence models of habitat suitability, which has been recommended for presence‐only models (Liu et al., 2005). After each individual model was created, we combined all models into a single combined spatial output (i.e., an ensemble model) to create a species richness layer of potential cheetah prey.

### Cheetah HSI

An alternative approach to SDM is the use of an HSI, where potential distribution is predicted using a numerical ranking of environmental conditions and resource availability and combined with an overall habitat suitability value for a selected species (Wakeley, 1988; Wildlife Service, 1981). Models of HSI have been used for decades and were some of the first suitability models to be widely employed by wildlife managers (Allen et al., 1988; Kliskey et al., 1999; Mitchell et al., 2002). Developing an HSI model requires knowledge of the relationship between a species and its habitat, particularly food and cover. This information is generally taken from published literature, determined through direct investigations, or compiled based on expert judgment (Brooks, 1997; Jarnevich et al., 2017).

Cheetahs, having once been widespread across most of Africa, the Middle East, and Central India, can survive and persist in a wide variety of landscapes (Durant et al., 2022; Rostro‐García et al., 2015). Other than tropical and montane forests, they occur in many different land‐cover types, including hyperarid desert, dense scrub, sparse vegetation, savannas, and woodlands (Ahmadi et al., 2017; Belbachir et al., 2015; Gros & Rejmanek, 1999). They generally avoid areas of human development (Cheraghi et al., 2018; Klaassen & Broekhuis, 2018) and are heavily reliant on the availability of prey species (Lindsey et al., 2011; Muntifering et al., 2006; Winterbach et al., 2015). Cheetahs commonly select prey weighing 23–56 kg, including small and medium antelope species (Hayward et al., 2006). However, their diet may include much smaller prey, such as hares (*Lepus* spp.), and species as large as greater kudu (*Tragelaphus strepsiceros*) when options are locally limited (Caro, 1994; Lindsey et al., 2011; Marker et al., 2003; Mills et al., 2004).

There is limited information on the conditions and habitat selection of cheetahs in our study area. Therefore, we elected to use an HSI model that included potential prey presence and richness, human modification, and crop cover (Table 1). The cheetah HSI was developed by first classifying areas that had high to moderate levels of human modification as unsuitable. Similarly, we then identified regions with >10% crop cover and classified those as unsuitable. Finally, we added the modeled prey species richness layer described above, retaining the richness value because we assumed that higher cheetah habitat suitability is correlated with higher prey species richness (Ferretti et al., 2020). These values and resulting model were informed by similar studies and expert opinion (Table 1). The final model had a spatial resolution of 1 km^2^ and provided a relative index of cheetah habitat suitability for the region that ranged from 0 (*least suitable*) to 7 (*most suitable*). We converted this to a binary layer of cheetah habitat suitability and unsuitability for the network model source data by performing a 90% kernel density estimate on the final HSI where values were >4. This identified the core areas of higher suitability that would likely support cheetah populations from which cubs may be taken illegally.

**TABLE 1:** Environmental variables used to develop the habitat suitability index (HSI) for cheetahs in the Horn of Africa.

Environmental variable	Description	Suitable classification threshold	Classification threshold references	Data source
Modeled prey species richness	Potential prey species habitat suitability models combined to represent prey species richness (0–7)	>0 species with increasing cheetah habitat suitability as species richness increases	Ahmadi et al., 2017; Hayward et al., 2006; Muntifering et al., 2006; Nazeri et al., 2015	Developed as a part of this work
European Space Agency (ESA) crop density	Proportion of a 1‐km^2^ area classified as crop cover based on 20‐m pixels	<10%; only low‐density areas of crops are suitable	Ahmadi et al. 2017; van der Meer, 2018	ESA CCI, 2017
Human modification	Human modification or landscape disturbance layer; continuous 0–1 metric that reflects proportion of a landscape modified based on modeling physical extents of 13 anthropogenic stressors and their estimated impacts	Expert opinion used to select only relatively low human modification areas to be designated as suitable (<0.25)	Cheraghi et al., 2018	Kennedy et al., 2020; Theobald et al., 2020

### Trafficking network models

We investigated how live cheetahs are moved from wild populations to known destinations. To do so, we built a spatially explicit model to identify probable trafficking routes (across land and water) connecting known and suspected wild cheetah populations with the pet markets and dealers in destination countries. Likely routes were identified using route optimization, which uses an algorithm to identify the shortest or most efficient routes between 2 locations. Route optimization presents a nontrivial challenge in large or complex transportation networks (Bast et al., 2016). However, in its simplest form, which we used, the strategies of finding the shortest path (i.e., shortest distance) between source and destination points and shortest travel time (accounting for variation in travel speed on different road/water routes) can provide meaningful information. We used multiple features of road networks, including road type, speed limits, and directionality from OpenStreetMap (https://www.geofabrik.de/), supplemented with manually created water routes, to determine optimal routes connecting source and destination nodes in a multimodal, multicontinent trafficking network. We then simulated likely trafficking routes between areas of known and modeled (i.e., HSI) cheetah populations in East Africa with georeferenced sites of confiscated trafficked cheetahs in East Africa or final destination locations identified from postings of cheetahs on social media platforms in the Arabian Peninsula.

We downloaded country‐level OpenStreetMap data from the Geofabrik website (https://www.geofabrik.de/) as.pbf files for each of our 21 study countries, and the road networks were extracted and saved as.osm files with the Osmosis command line tool (Osmosis Contributors, 2023). OpenStreetMap data are not always complete, accurate, or recent. For all the countries in our study, we extracted roads to the level of secondary highways, which typically are not major routes in a country but constitute links to large roads (e.g., 2‐lane roads, arterial roads in cities, etc.). However, in Eritrea and Somalia with sparse road networks, tertiary roads were also included to better capture connectivity between towns, the road networks of adjacent countries, and port locations. Road network files for all countries were merged into a single data set of merged roadways.

Potential water crossings from East Africa to the Arabian Peninsula were manually created in QGIS (QGIS Development Team, 2022), connecting nodes at known port locations and nodes closest to coastlines in Africa with similar nodes in the Arabian Peninsula, and merged with the road network data set.

Travel speed for roads was set by OpenStreetMap data (i.e., dependent on road type) and for marine and freshwater routes at the estimated speed of wooden *dhows* that are commonly used for trafficking activities (Sayehbani & Zeraatgar, 2005). In OpenStreetMap data, complex intersections and roundabouts often contain large numbers of nodes that were not necessary for capturing potential transportation routes and that greatly increased the size of the data and thus computation time. The OSMnx Python (Boeing, 2017) and NetworkX (Hagberg et al., 2008) packages were used to simplify the merged transportation network by removing unnecessary nodes and edges while preserving the network topology.

Approximately 100 point locations were randomly generated in each of the cheetah habitat polygons (i.e., known and HSI model) to simulate potential origin locations. For each point, the nearest node in the full transportation network was calculated and used as the source or origin node for entry into the road network. Destination nodes (*n* = 34) were known locations, including from social media posts with identifiable locations, identified in Tricorache et al. (2021) as locations where cheetah cubs were being sold or identified as pets. We also included the Red Sea and Gulf of Aden as part of the maritime trade route. These traditional and historic routes between the Horn of Africa and the Arabian Peninsula are still regularly used today for trade and transport of a wide variety of goods, including livestock, charcoal, food, fuel, frankincense, arms, and people (Majid & Abdirahman, 2019). We used Dijkstra's algorithm (Dijkstra, 1959) to calculate the shortest path from each of these origin nodes to each of the destination nodes for both distance and travel time. Travel time was estimated for every edge in the transportation network as the product of the edge length and the estimated speed limit for the given edge type as provided in the OpenStreetMap data (e.g., major highways, smaller urban streets) and water routes (∼20 knots [Sayehbani & Zeraatgar, 2005]). The OSMnx Python library further allows for calculating *N* optimal routes between source and destination nodes. Qualitative analyses of potential bottleneck points were performed with density plots to visualize the spatial concentrations of optimal routes at node locations (e.g., ports, border crossings, cities).

## RESULTS

### HSI models

**FIGURE 3:**
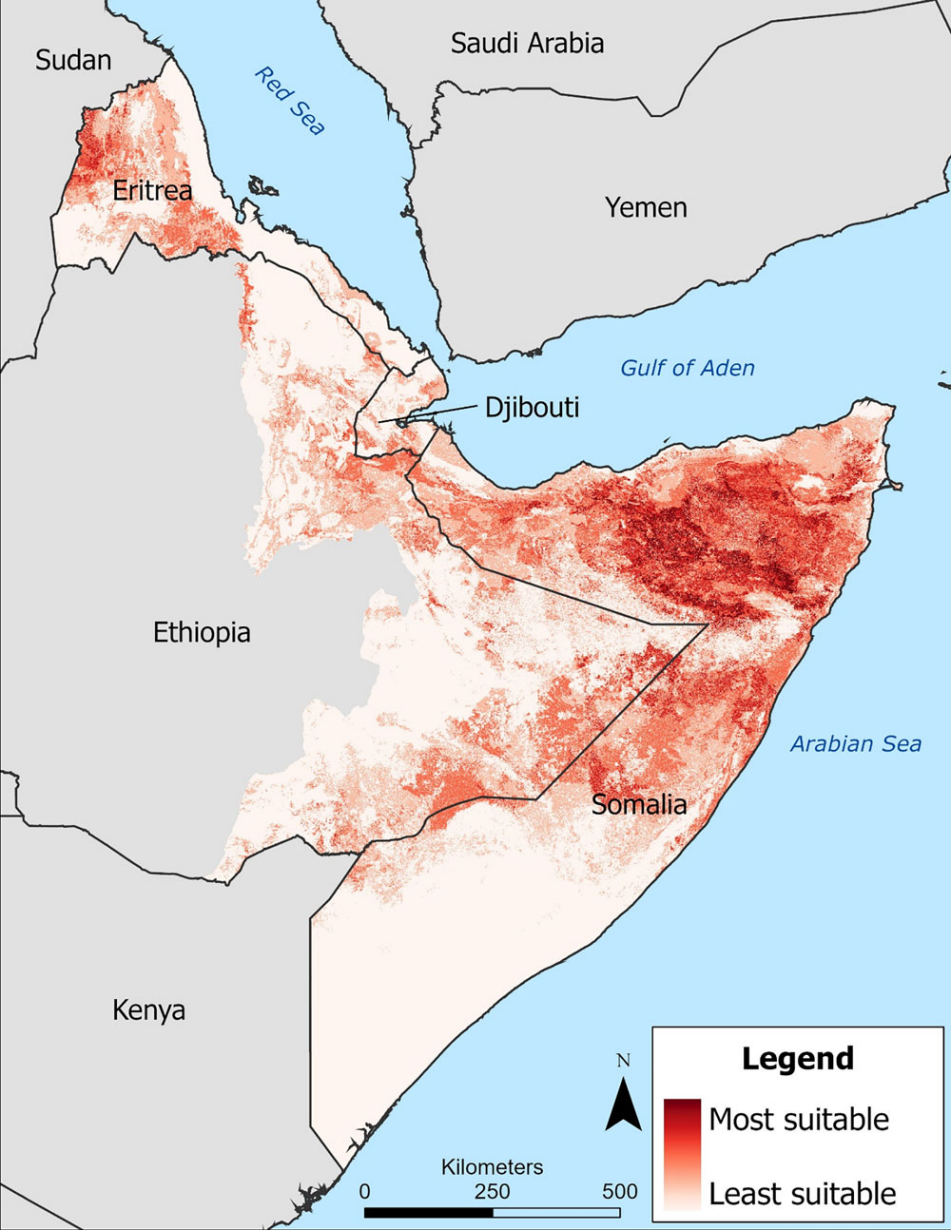
Cheetah habitat suitability index (HSI) model showing relative suitability of cheetah habitat.

All cheetah prey suitability models performed well with test AUC values >0.70 except for *L. walleri*, *M. guentheri*, and *M. saltiana*, which had test AUCs >0.60 because these species are generalist species, which directly reflects the predictive accuracy of habitat suitability models (Evangelista et al., 2008). The cheetah HSI identified approximately 892,000 km^2^ (76% of the study area) of habitat that could support cheetah (Figure 3). Regions of the study area that were predicted to be the most suitable for cheetahs included central and eastern Somaliland, the border between the Somali Regional State in Ethiopia and Somalia, and the central region of Puntland and northwestern Eritrea. Recent observations, reports, and publications have documented cheetahs in many of the areas in our HSI. These include interviews in pastoral communities throughout Somaliland (Evangelista et al., 2018) and Somali Regional State in Ethiopia (Abdella et al., 2023), recently confirmed sightings of cheetahs in the Awdal region of Somaliland (Marker et al., 2023), camera trap photos in the Digri Plateau of Djibouti (Murgatroyd et al., 2023), and others including those highlighted in Figure 1. These confirmed observations support our cheetah HSI predictions, indicating that much of this area is suitable for cheetahs and populations occurred in multiple locations throughout the region.

### Trafficking network models

**FIGURE 4:**
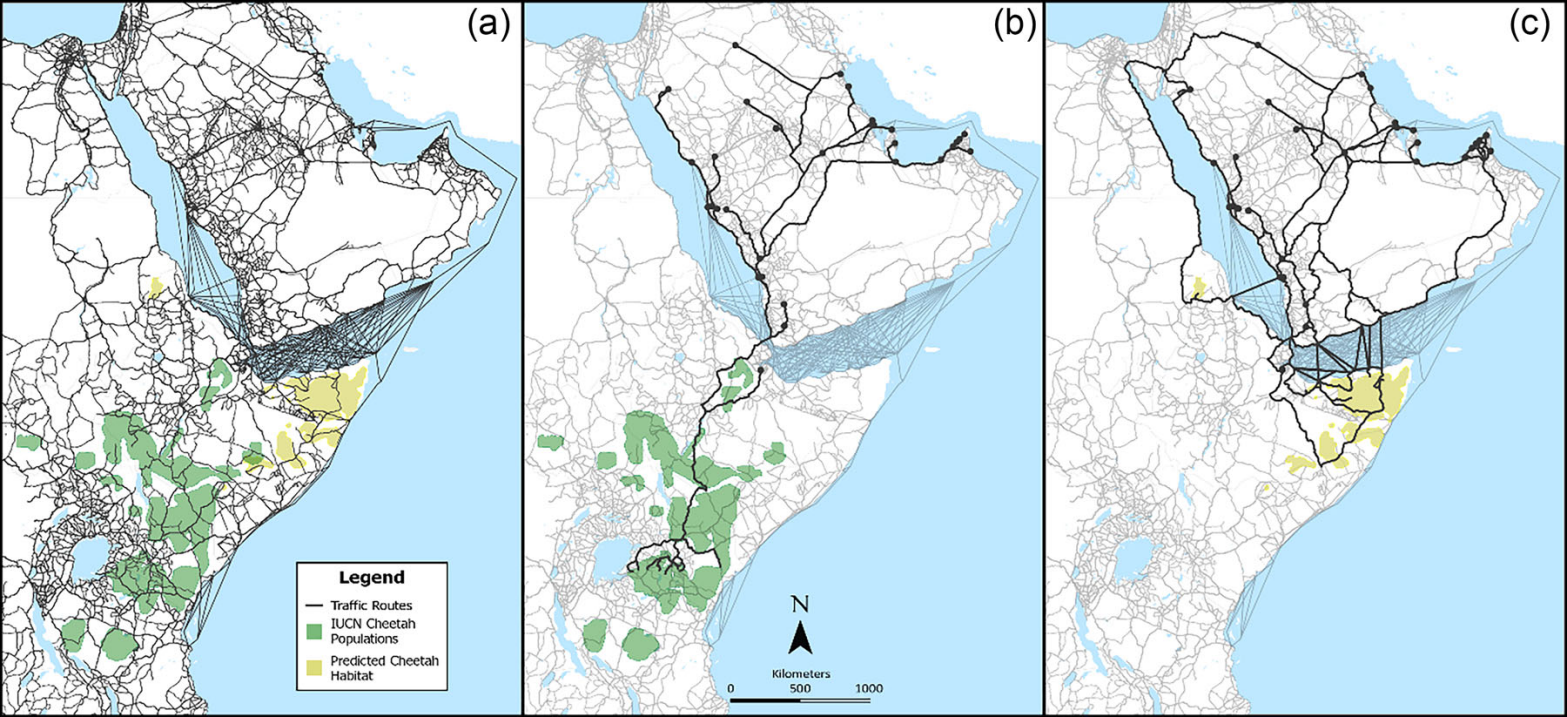
Model outputs for (a) potential trafficking network routes from OpenStreetMap augmented by digitizing shipping routes, (b) predicted trafficking networks from a sample of known cheetah populations from the International Union for Conservation of Nature (Durant et al., 2017) to known destinations (Tricorache et al., 2021), and (c) trafficking network models from potential undocumented cheetah populations estimated from our habitat suitability index (HSI) to known destinations.

The original OSMnx graph constructed from OpenStreetMap data and the manually created water routes consisted of over 202,000 nodes. The simplified and consolidated network used for modeling consisted of approximately 87,000 nodes. Similarly, the original graph consisted of approximately 331,000 edges, whereas the simplified and consolidated network contained approximately 165,000 edges (Figure 4a).

As such, the network used for modeling represents an abstracted version of actual road and water routes, and attributes such as speed limits and lengths belong to the simplified network rather than being represented in the spatial geometries of the roads and water routes. This had an impact on the number of sampled origin locations in that multiple randomly generated point locations in habitat polygons often snapped to the same nearest node in the transportation network. This was particularly prevalent in areas with sparse roads or nodes. As a result, the modeling incorporated approximately 760 origin locations in the known cheetah habitat polygons and 38 origins in the modeled habitat locations. In effect, these origins represented the nearest access point to the transportation network.

**FIGURE 5:**
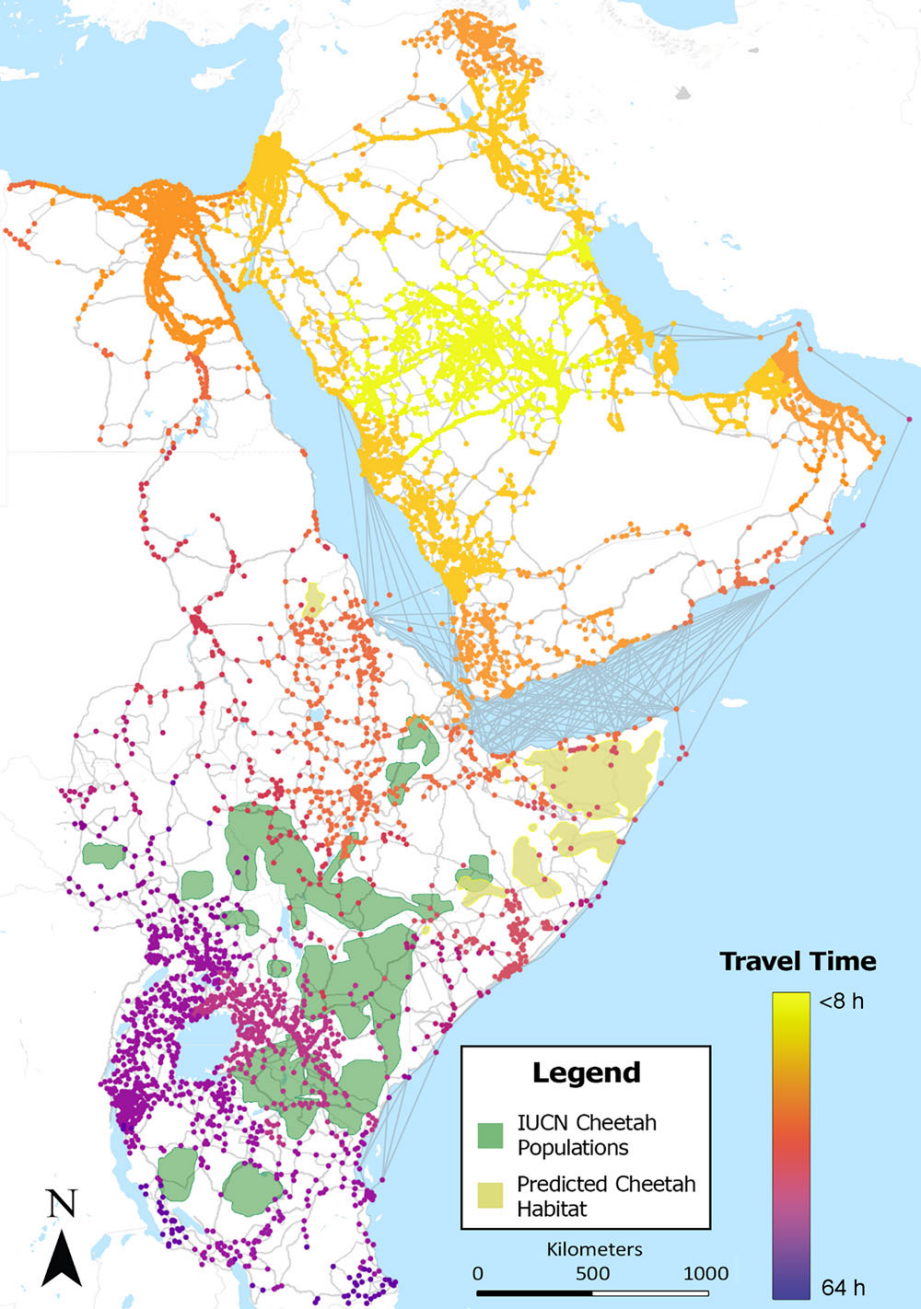
Isochrone model output showing travel time in hours from a known destination location (center of Arabian Peninsula, bright yellow) to known cheetah populations from International Union for Conservation of Nature (green) and our cheetah habitat suitability index (tan).

**FIGURE 6:**
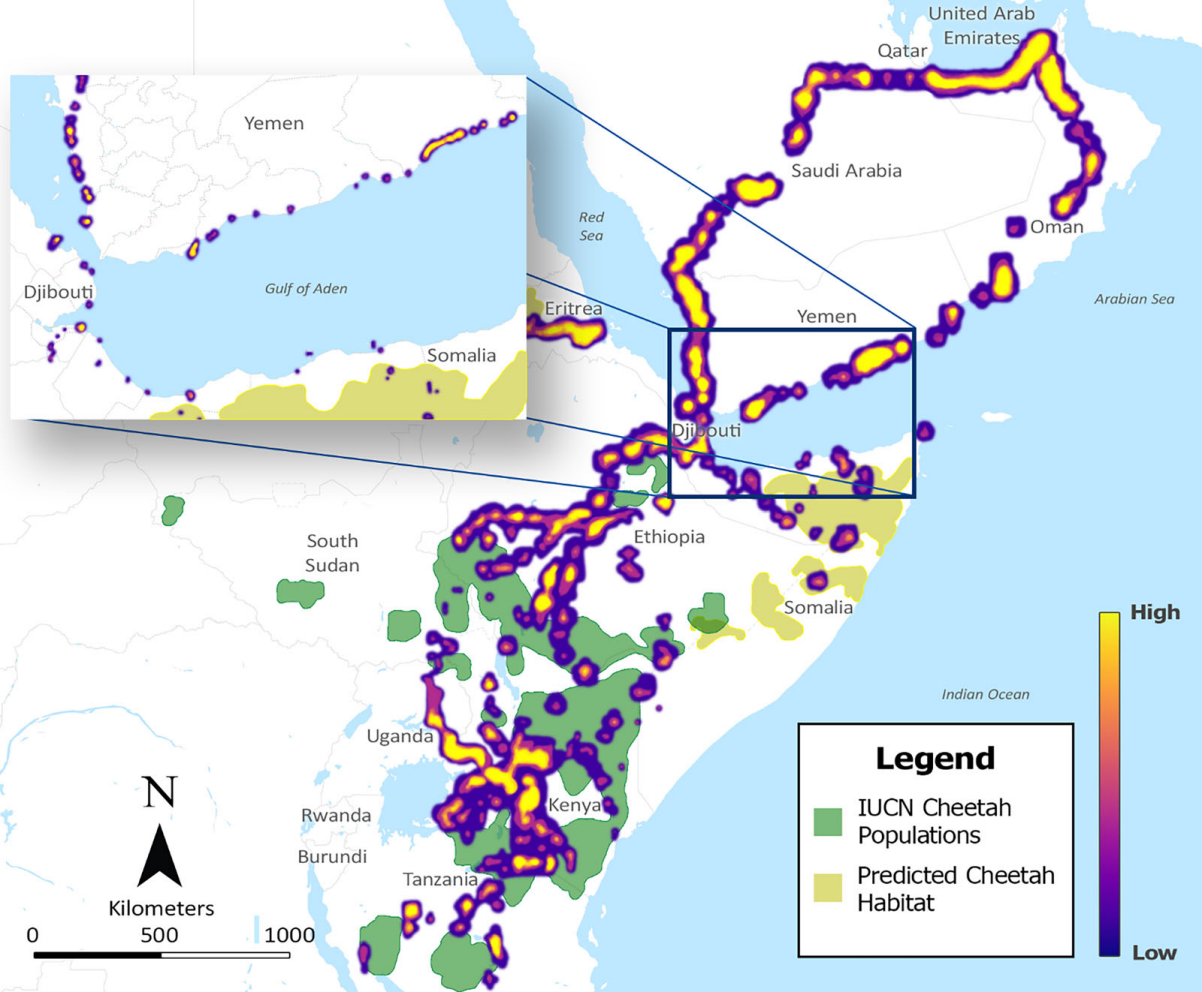
Cheetah trafficking network model output showing towns and ports where multiple routes intersect or are concentrated.

The simplified network and origin and destination points were used to model scenarios, such as all origin points in a given polygon to all known destinations (Figure 4b) and isolating a single origin and mapping routes to all known destinations (Figure 4c). OSMnx also allowed for the creation of isochrone maps in which a single destination point could be selected and every node in the network is depicted by colors representing different travel times to and from that destination (Figure 5, scenario of 8‐h travel days from a single destination). We further explored the likely routes from East Africa to the Arabian Peninsula by constructing an interactive map of node locations in a heatmap to identify node locations with higher numbers of potential routes crossing through them (Figure 6). The Horn of Africa, particularly Somaliland, Puntland, and the Somali Regional State of Ethiopia, had shortest travel times to end destinations. Primary road networks appeared important to the trade, and some key nodes where law enforcement interventions might be particularly effective were identified (Figure 6).

## DISCUSSION

The inherent complexities of IWT (spanning multiple scales, sectors, and many species‐specific factors) warrant novel approaches that engage in “convergent science,” crossing currently siloed disciplinary approaches and further employing quantitative modeling to advance understanding of IWT (Gore et al., 2023). Our stepwise approach furthers such efforts in that it combines the power of HSI with network modeling. Our case study of live cheetah trade shows how the approach can be used to address key information gaps and inform conservation interventions. However, our results must be applied with caution because validation data for our HSI and trafficking network models are sparse due to the inaccessibility and remoteness of the region. Furthermore, there are several caveats that need to be considered when interpreting the models’ utility and results.

Species distribution modeling has been used to determine habitat selection and the extent of cheetah range. Most of these models relied heavily on vegetation structure or topography and varied anthropogenic influences, such as roads or land use, and generally did not adequately consider the availability of prey species (Klaassen & Broekhuis, 2018; Kuloba et al., 2015; Shams‐Esfandabad et al., 2021; Tagwireyi et al., 2020). Although we integrated the presence of multiple prey species in our HSI, prey richness did not equal prey availability or abundance. Though the results of several studies suggest that prey richness can enhance predator populations (Ferretti et al., 2020; Sandom et al., 2013), including cheetahs (Ahmadi et al., 2017), it is widely believed that cheetahs are more likely to respond to abundance than richness. Still, our results offer a useful way to parameterize network models and provide an initial guide to conduct more intensive surveys.

Our trafficking network model relied heavily on road networks from OpenStreetMap. These data are derived from crowdsourced information, which depends on a wide network of data contributors who rarely have direct knowledge of remote or rural areas in developing countries, particularly in areas experiencing conflict or political insecurity. Although we found that OpenStreetMap offered the best available data for our model development, it is likely there are errors and the data are incomplete. Furthermore, there are more traditional means of transport, such as camels and donkeys, and cross‐country routes, that are not accounted for. The border between Ethiopia and Somalia, for example, is approximately 1600‐km long and porous. Undoubtedly, there is much unofficial trade along unconventional routes between the 2 countries due to the shared ethnic, linguistic, and religious characteristics. Furthermore, poor integration of borderlands with the state governments facilitates official and unofficial trade across the Ethio‐Somali border, including livestock, agricultural products, charcoal, chat (or khat), and contraband (Tekan & Azeze, 2002). In the case of cheetahs, however, the only recorded instance where cubs were transported along an unconventional road network was an incident involving 3 cheetah cubs carried by camel across the border in 2013, from the Hadigud area in Ethiopia to Daradere in Somaliland, after the vehicle being used broke down in the bush (Tricorache et al., 2021). We assumed that although cheetahs and other wildlife are likely transported by camels, donkeys, or other means on short journeys, any long‐distance transport of cheetah cubs in this manner is not practical or economical given how fragile they are and the increased risk of death during transport.

Despite their shortcomings, we believe our models provide an understanding of cheetah trafficking networks and identify undocumented populations. With the increase in global positioning system (GPS) technologies and open‐source travel data, such as air travel routes and OpenStreetMap, travel network modeling is emerging as a promising tool in combating IWT (Ferber et al., 2023). Such models are used commonly in transportation and logistics planning as methods for determining optimal routes between locations (Fox, 2018). Our analysis of potential trafficking networks and transportation routes used in IWT of cheetahs can inform the next steps to address the supply and demand of live cheetah cubs.

Related to the supply side, most current IWT approaches focus on law enforcement, and our tool can benefit these efforts, particularly in evaluating potential deterrence strategies, such as targeted border closures and police checkpoints, at identified transit hotspots (Gore et al., 2021; Magliocca et al., 2024). Many transit routes likely occur through open borders where there is minimal to no government presence (Figures 5 & 6). This suggests that traffickers are already utilizing areas where law enforcement is minimal and that nontraditional approaches are required to reduce trafficking of live cheetahs and other wildlife.

Our trafficking network model can also be used to develop capacity among state government partners, intergovernmental bodies (e.g., Intergovernmental Authority on Development/Horn of Africa Wildlife Enforcement Network), range state academic institutions, and nongovernmental organizations working to reduce illegal trade in the cheetahs’ range. Given limitations in law enforcement and legal measures to reduce the supply of wildlife without addressing the underlying socioeconomic and governance conditions in source locations (Browne et al., 2021), there is an urgent need to improve community collaboration and engagement in addressing IWT. This ranges from increasing general awareness about cheetahs, understanding community attitudes and beliefs about cheetahs and trafficking, cocreating solutions with communities that address their concerns related to cheetahs and other wildlife, and equitably sharing benefits and management decisions about cheetahs with local communities. There is also utility in using this tool, especially the HSI maps, to target areas where increased and improved community engagement is desperately needed in the Horn of Africa to build a local constituency for cheetah conservation. Demand reduction efforts can also be informed with this tool. Most strategies for demand reduction require significant investments of time and funding (Browne et al., 2021), and this tool can be used to identify areas where limited resources can be focused more efficiently. These applications directly support the needed conservation actions highlighted by the IUCN Red List of Threatened Species (Durant, Broekhuis, et al., 2023).

Although the importance of integrative, quantitative tools to advance knowledge and action on IWT is increasingly recognized (Gore et al., 2023; Keskin et al., 2023), it is essential to ensure these modeling efforts are not standalone or one‐off exercises. In addition to building the capacity of wildlife management actors to use these tools to more holistically approach IWT and cheetah conservation regarding law enforcement, community outreach, and demand reduction strategies, model results should be used to inform biological monitoring and field validation exercises that explicitly engage local communities. Specifically, model results can be used to target areas for additional field validation of cheetah occurrence and data collection efforts that further center on engagement with local communities. Regarding the latter, approaches, such as mental models and risk mapping, are needed to engage local communities that rely on these shared landscapes to better comprehend their collective understanding of IWT and their interactions with cheetahs and to design interventions that address their diverse needs (e.g., Gore et al., 2023; Moon et al., 2019). This engagement should be facilitated through meaningful, participatory approaches centered on knowledge coproduction and “reflective inquiry” that seeks to empower local voices, enhance collaboration, and improve equitable outcomes (Laituri et al., 2023) because these are the stakeholders who will ultimately determine the fate of cheetahs in the region.

## Supporting information




**Appendix S1**. Table of Environmental variables representing climatic conditions, landcover type, human modification, and topography.
