# Supplementary material for: Mapping illegal trade routes of live cheetahs from the Horn of Africa to the Arabian Peninsula
Source: Conserv Biol. 2024 Oct 25;39(3):e14412. doi: 10.1111/cobi.14412 (PMC12124172; doi:10.1111/cobi.14412)
Supplement: Supplementary file 1 — Appendix S1. Table of Environmental variables representing climatic conditions, landcover type, human modification, and topography. [file COBI-39-e14412-s001.docx]

**SUPPORTING INFORMATION**

**Appendix S1.** Table of Environmental variables representing climatic conditions, landcover type, human modification, and topography.

| **Environmental variable** | **Details** | **Source** |
| --- | --- | --- |
| Land cover | 20-meter land cover product with 10 cover classes for all of Africa. Seven of the total ten classes (water, snow/ice, and spare/alpine vegetation were not included due to their limited occurrence in the study area) were converted to a continuous variable representing the proportion (percent) of the landscape that is occupied by a specific land cover type within a 1km^2^ area using a moving window analysis. This resulted in seven variables for each land cover type. | European Space Agency CCI Land Cover team (ESA CCI 2017) |
| Climate | We used six Bioclimatic variables from WorldClim 2 that were selected based on expert opinion. These included: Bio 1 (Annual mean temperature), Bio 8 (Mean temperature of wettest quarter), Bio 9 (Mean temperature of direst quarter), Bio 12 (Annual precipitation), Bio 16 (Precipitation of wettest quarter), and Bio 17 (Precipitation of driest quarter). | WorldClim v2 (Fick and Hijmans 2017) |
| Topography | Elevation, slope, northness, eastness | SRTM elevation data (Farr and Kobrick, 2000) |
| Average annual NDVI | Average annual Normalized Difference Vegetation Index (NDVI) across 20 years (2000-2020 | Based on Landsat imagery and analyzed in Google Earth Engine (Gorelick et al. 2017) |
| Human modification | Human modification or landscape disturbance layer with continuous 0-1 metric that reflects the proportion of a landscape modified, based on modeling the physical extents of 13 anthropogenic stressors and their estimated impacts for 2017. | Theobald et al. 2020. |
